# Exceptionally small supramolecular hydrogelators based on aromatic–aromatic interactions

**DOI:** 10.3762/bjoc.7.23

**Published:** 2011-02-07

**Authors:** Junfeng Shi, Yuan Gao, Zhimou Yang, Bing Xu

**Affiliations:** 1 Department of Chemistry, Brandeis University, 415 South Street, Waltham, Massachusetts 02453; 2 The Key Laboratory of Bioactive Materials, Ministry of Education, College of Life Science, NanKai University, Tianjin 300071, People’s Republic of China

**Keywords:** aromatic–aromatic interaction, cinnamoyl, hydrogel, hydrogelator, supramolecular

## Abstract

We report herein the use of an aromatic–aromatic interaction to produce small molecule hydrogelators that self-assemble in water and form molecular nanofibers in the resulting hydrogels. Among these hydrogelators, a hydrogelator (**6**) made from a phenylalanine and a cinnamoyl group represents the lowest molecular weight (MW = 295.33 g/mol) peptide-based hydrogelator prepared to date. The supramolecular hydrogels were characterized by transmission electron micrograph (TEM) and fluorescence spectroscopy, and the results obtained by both techniques correlate well with their rheological properties. Notably, compound **6** can undergo *cis*/*trans*-isomerization upon UV irradiation.

## Introduction

Gels formed by three-dimensional, elastic networks to encapsulate a liquid [1], have many useful properties (e.g., response to external stimuli and flow in response to shear force [2]) and applications in several areas (e.g., bioanalysis [3–4], chemical sensing [5–7], food processing [8], cosmetics [9], drug delivery [10–11], and tissue engineering [12–13]). Inspired by the existing and potential applications of gel materials, research on supramolecular gels [14–19] has rapidly expanded. Amongst these, self-assembled oligopeptides [20–23], which self-assemble in water to form nanofibers and provide hydrogels for biomedical applications, have stimulated the recent research efforts on low molecular weight hydrogelators [24–29]. Despite the intensive research and rapid advances in the design and synthesis of low molecular weight hydrogelators, the minimum structural requirement for a small molecule to act as a hydrogelator to form supramolecular hydrogels has been less explored. We have shown that aromatic–aromatic interactions induce the self-assembly of glycopeptides [30] or pentapeptidic derivatives [31] in water to form nanofibers and supramolecular hydrogels. These results, together with the supramolecular hydrogelators made from dipeptide conjugates with fluorenyl or naphthyl groups, clearly support the simple notion that aromatic–aromatic interactions in water may direct the formation of hydrogen bonding and would be useful for the supramolecular self-assembly of small molecules in water.

However, despite the progress noted above, an important question still remains to be answered: What is the minimal set of aromatic–aromatic interactions and hydrogen bonding for molecular self-assembly in water to produce supramolecular nanofibers and hydrogels? About a decade ago, Menger [32] and coworkers showed that an aroyl L-cystine derivative, which has a molecular weight as low as 448.51 g/mol, can form a hydrogel at a concentration as low as 0.2 mM and elucidated the molecular structure of the hydrogel based on the crystal structure of the gelator. During that gelation experiment, dimethyl sulfoxide (DMSO) was required in the gel, however, this might complicate the accurate assessment of the aromatic–aromatic interactions and hydrogen bonding since DMSO is prone to form hydrogen bonds with hydrogen bond donors. Therefore, it is necessary to explore small supramolecular hydrogelators for correlating the molecular structure (e.g., numbers of hydrogen bond donors and acceptors and aromatic groups) and the capability of self-assembly without the inclusion of DMSO. Recently, Dastidar and Das [33] reported that *N*-(4-pyridyl)isonicotinamide (MW = 199.21 g/mol) can act as a non-polymeric hydrogelator at the concentration of 0.37 wt %, which further suggests the possibility of peptide-based hydrogelators that are smaller than the aroyl L-cystine noted above.

Here, we reported the systematic synthesis and examination of a series of phenylalanine derivatives and the identification of the lowest molecular weight peptide-based hydrogelator (MW = 295.33 g/mol) produced to date. Since phenylalanine derivatives belong to a class of insulin absorption promoters [34], this work not only provides a possible benchmark for low-molecular weight hydrogelators, but also a simple system to help in understanding the self-assembly of these phenylalanine based molecules in water and this may offer insights to address side effects, polymorphrism, and efficacy of drug candidates that share common molecular features with these hydrogelators.

## Results and Discussion

**Scheme 1:**
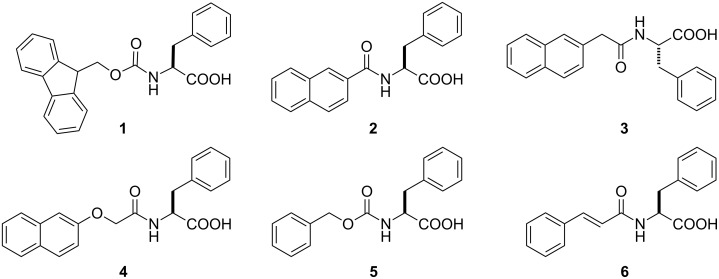
The chemical structures of the phenylalanine derivatives.

Scheme 1 shows the structures of the phenylalanines investigated in this work. We chose four types of aromatic moiety, fluorenyl, naphthyl, naphthalenoxyl, and cinnamoyl groups, to covalently attach to the phenylalanine via a simple amide bond. On treatment with 1N NaOH solution, all the prepared compounds dissolve. However, when the pH of the solution is changed from basic to slightly acidic, compounds **1**, **3**, **4** and **6** form hydrogels. Compared with compound **3**, compound **2** fails to form a gel due to the conjugation between the carbonyl group and the naphthyl group, which partial reduces the rotational freedom of the naphthyl group that is necessary for self-assembly. As shown in Table 1, all gels (gels **I**, **II**, **III** and **IV** formed by compounds **1**, **3**, **4** and **6**, respectively) are thermally and pH reversible. For example, heating the gel formed by 0.3 wt % of compound **1** in water to 56 °C or changing its pH from 6.6 to 9.0 leads to a gel–sol phase transition. The gel forms again after restoring the previous conditions, and this cycle can be repeated several times.

**Table 1:** Typical conditions for the hydrogelation of the phenylalanine derivatives.

Gel#	Compound	Conc. (wt %)^a^	pH^b^	Temp. (°C)^b^	γ_0_ (%)^c^	*G*_0_ (Pa)^d^

**I**	**1**	0.3	6.6	55	0.20	50199
**II**	**3**	0.5	5.7	45	0.26	4849
**III**	**4**	0.7	5.9	48	0.56	7820
**VI**	**6**	1.0	4.6	41	0.85	2519

^a^The minimum concentration of the gelator needed for gelation, ^b^the gel–sol phase transition pH/temperature at concentration of 1.0%, ^c^critical strain [35], ^d^elastic constant.

**Figure 1:**
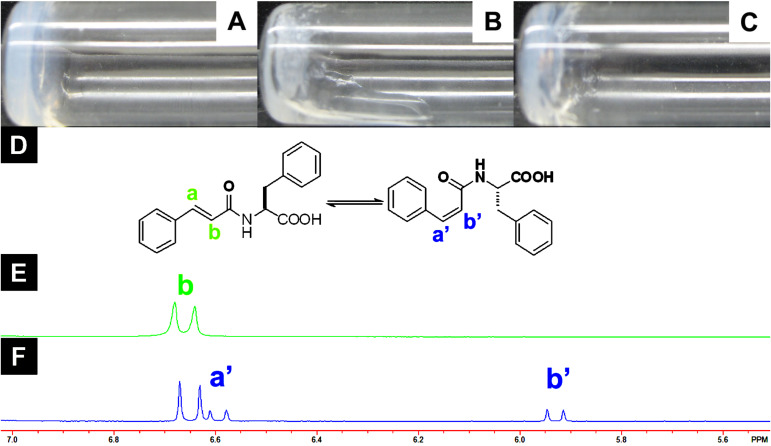
Optical images of (A) gel **IV** (1.5 wt %, pH = 4.6), (B) gel **IV** after UV irradiation (no aging), (C) gel **IV** after UV irradiation (aging 2 hours), (D) the structure of compound **6** in *trans*- and *cis*-isomers**,** (E) the NMR spectra of compound **6** before and (F) after UV irradiation for 2 hours.

We noted that the gel **IV** exists in two phases (Figure 1B) when it was irradiated by 254 nm UV light for 2 hours: One phase appears solid like, whilst the other phase flows. Two layers of gels are formed after 2 hours aging (Figure 1C). In order to understand the reason for phase separation, compound **6** was placed in a UV reactor for the same duration. As revealed by the ^1^H NMR (Figure 1F), a new set of peaks at 5.9 ppm and 6.6 ppm appears after UV irradiation, indicating that *cis*-**6** forms upon the photo-excitation [36]. Since there was no new peak in the range of 2.5 to 2.9 ppm (Figure S1, Supporting Information File 1), photopolymerization or photodimerization are unlikely. Furthermore, the result shown in Figure S2 (Supporting Information File S2) also supports the contention that heating is insufficient to cause conversion of the *trans*-isomer to the *cis*-isomer during UV irradiation. Thus, we infer that the formation of two layers in the gel **IV** after UV irradiation originates from geometric (*cis*/*trans*-) isomerism.

**Figure 2:**
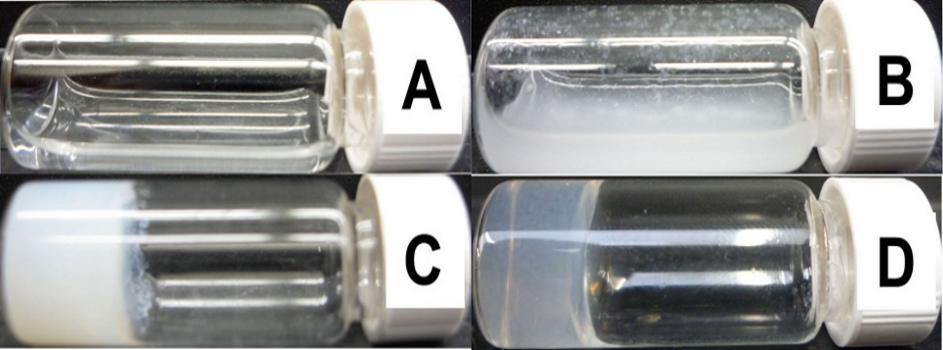
The optical images of (A) solution of **6** (2 wt %, pH = 9.0), (B) suspension of **6** (2 wt %, pH = 6.5), (C) opaque gel of **6** (2 wt %, pH = 4.6), (D) transparent gel of **6** (2 wt %, pH = 4.6, aging for 10 days).

Compound **6** also exhibits another interesting phenomenon. On acidification by 1N HCl solution, the solution of **6** (Figure 2A, 2.0 wt %) first turns from a clear solution into a suspension (Figure 2B). The suspension becomes an opaque gel (Figure 2C) when the pH reaches 4.6, which finally turns into a clear hydrogel after aging for ten days (Figure 2D): The aging process apparently helps the small molecules of **6** to disperse evenly and self-assemble again. This behavior seems unique to **6** in these phenylalanine derivatives because solutions of the other compounds on acidification change directly to either gels or suspensions, and the suspensions are unable to become gels spontaneously. This interesting gelation process suggests that the reversible *cis*/*trans*-isomerization provides an additional pathway for rearranging the supramolecular structures and achieving ordered supramolecular structures to produce well-dispersed nanofibers as the matrices of the hydrogels. The fact that compound **6** has the lowest molecular weight amongst the peptide-based hydrogelators suggests that a single amino acid molecule is unlikely to be able to provide an adequate amount of supramolecular interactions to form nanofibers, entrap the solvent, and result in hydrogelation. Apparently, the cinnamoyl group is the minimum structure motif to provide sufficient aromatic–aromatic interactions for a phenylalanine derivative to act as a hydrogelator.

**Figure 3:**
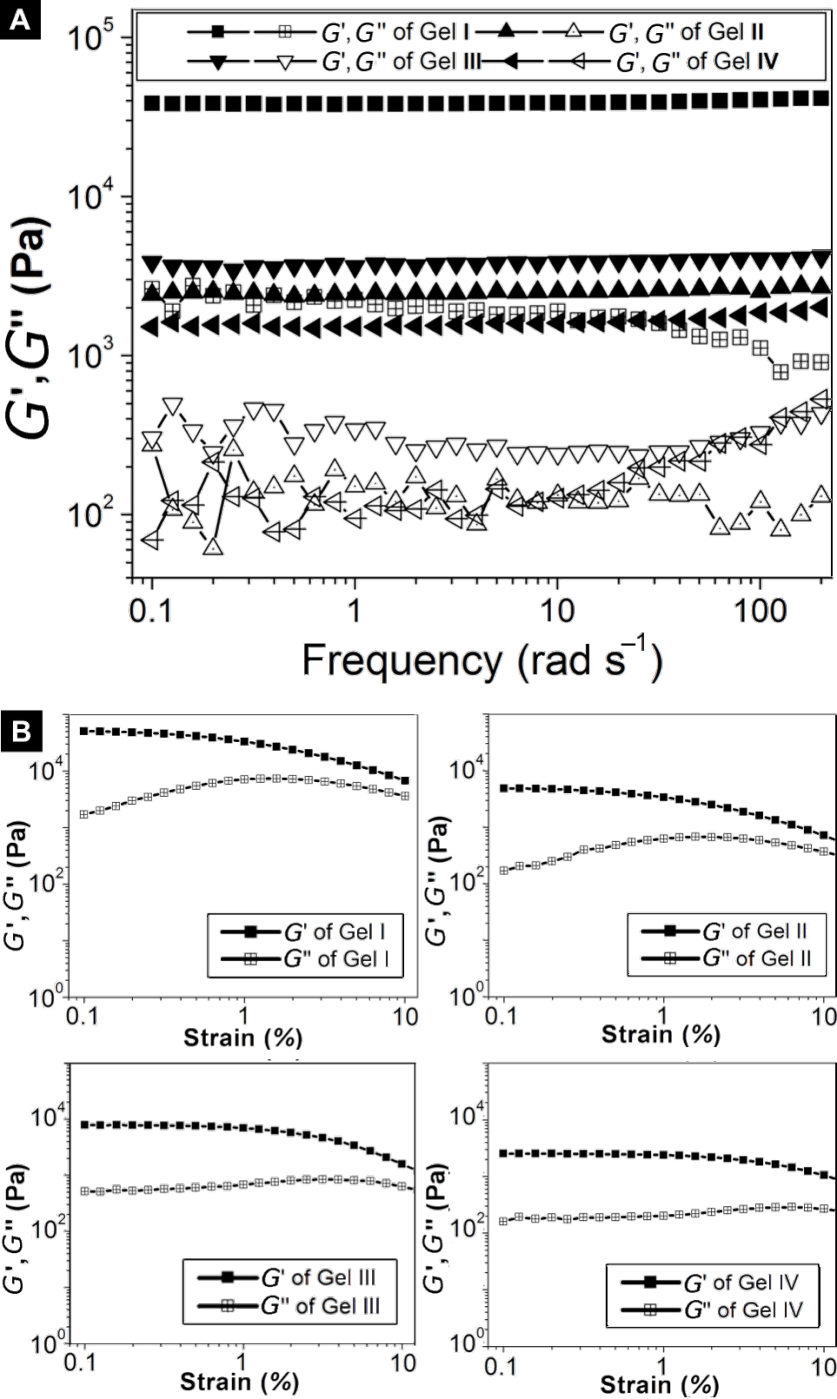
(A) Frequency dependence of dynamic storage modulus (*G*’) and loss modulus (*G*”) of gels **I** to **IV** at 1.0 wt % concentration. (B) Dynamic storage modulus (*G*’) and loss modulus (*G*’’) versus strain for the gels **I** to **IV**.

**Figure 4:**
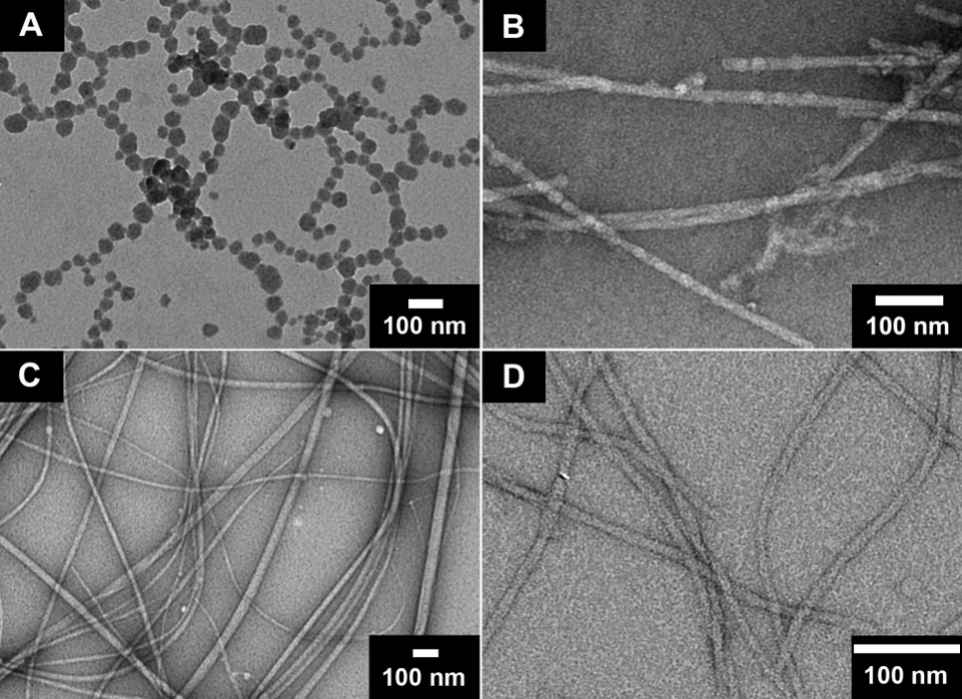
TEM images of the nanofibers that act as the matrices of gel **I** (A), gel **II** (B), gel **III** (C) and gel **IV** (D).

Figure 3 shows the frequency dependence of storage modulus (*G*’) and loss modulus (*G*’’) for gels **I** to **IV** at a concentration of 1.0 wt %. The values of the dynamic storage moduli (*G*’) of all gels are higher than those of their dynamic loss moduli (*G*”), indicating that all samples behave as viscoelastic materials. The values of *G*’ of the hydrogels exhibit little dependence on the frequency (from 0.1 to 200 rad/s), suggesting that the matrices of gels have good tolerance to external shear force. Gel **IV**, which formed by the lowest molecular weight hydrogelator among the phenylalanine derivatives prepared, has the lowest storage modulus. Gel **I**, which was formed by a hydrogelator with the highest molecular weight among compounds **1**–**6**, exhibits the strongest mechanical strength among the gels reported in this work. This is in agreement with the fact that the interactions between fluorenyl and phenyl groups are stronger than the interactions among phenyl groups. Figure 3B shows the modulus (*G*’ and *G*’’)-strain profiles of gels **I** to **IV**, which provide the maximum *G*’ values in the linear region and values of critical strain. The *G*’ remained almost constant when the strain increased and then suddenly decreased, indicating the loss of crosslinking within the gel network. Obviously, the critical strain of gel **IV** is greater than the others. These results suggest that gel **IV** can form a more complex network structure, which agrees with the morphology as revealed by TEM (Figure 4).

Figure 4 shows the TEM images of the matrices of gels **I** to **IV**. The phenylalanine based hydrogelator self-assemble into nanofibers that physically cross-link to form a fibrous network as the matrix of hydrogel. For example, the fibers in gel **III** (Figure 4C) are longer and larger than those in gel **II** (Figure 4B), agreeing with the fact that gel **III** has a larger critical strain and higher storage modulus. In the TEM image of gel **IV** (Figure 4D), the fibers are smaller and longer than other gels, which could contribute to its relative high critical strain and low storage module values. Unlike other gels, nanoparticles (20–80 nm) string together to constitute the matrices for gel **I**, indicating a high tendency for aggregation. This result also supports the fact that the fluorenyl and phenyl groups have the strongest interactions among those hydrogelators and result in the highest storage moduli among the gels **I**–**IV**.

**Figure 5:**
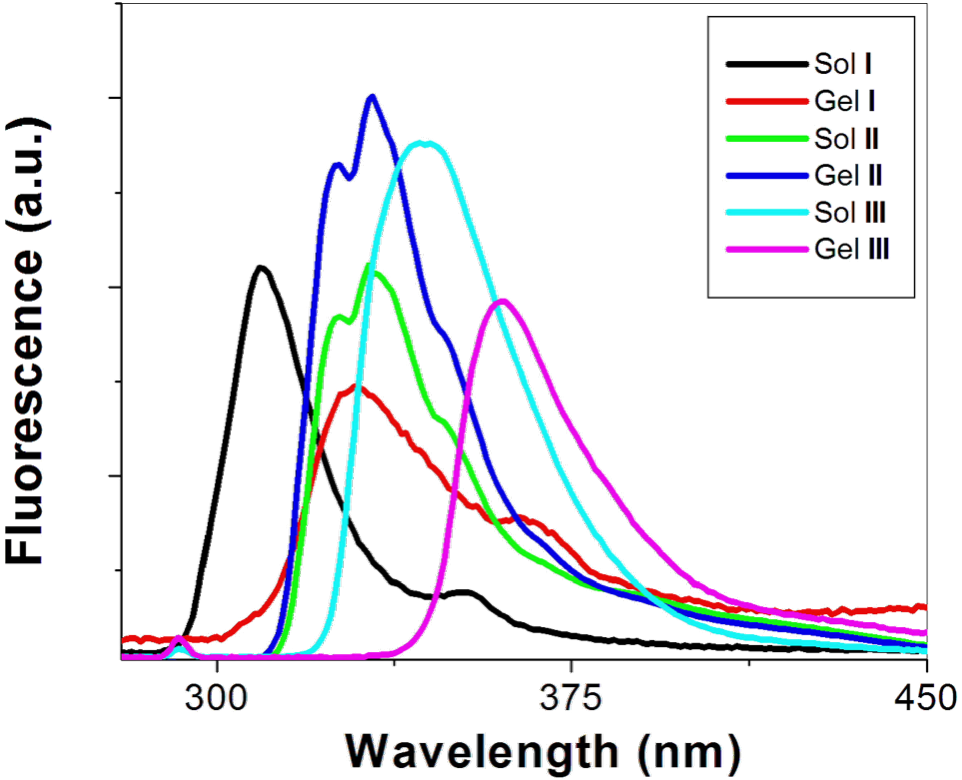
The emission spectra (slit width = 3.0 nm) of the gels **I**–**III** and their solutions (**I**: λ_ex_= 265 nm; **II**, **III**: λ_ex_= 292 nm).

Based on emission spectra of the hydrogels (Figure 5), aromatic–aromatic interactions increase in the gel state, evidenced by the fact that most of bands in the gel phase show red shift. In gel **I**, the band centers at about 309 nm in solution and shift to about 329 nm in the gel phase, suggesting that Fmoc group overlaps with the phenyl group; the shoulder at 363 nm likely originates from the antiparallel dimerization of the fluorenyl group whilst the small broad bands above 400 nm apparently relate to trimeric or tetrameric aggregates of the Fmoc groups. In gel **II** and gel **III**, the red shifts are smaller than that of gel **I** (from 332 to 333 nm in gel **II**, from 345 to 360 nm in gel **III**), suggesting that the interactions between naphthyl groups and phenyl groups in compounds **3** and **4** result in less overlap than that of fluorenyl and phenyl groups.

## Conclusion

In summary, we have demonstrated that an aromatic group (fluorenyl, naphthyl, naphthalenoxyl, or cinnamoyl) covalently attached to phenylalanine gives rise to a series of new low molecular weight hydrogelators and the identification of the lowest molecular weight peptide-based hydrogelator prepared to date. Obviously, the cinnamoyl group is the minimum structure motif to provide sufficient aromatic–aromatic interactions for a phenylalanine derivative to be a hydrogelator. It not only provides a useful experimental system for further elucidating the relationship between hydrogelation and molecular structure, but also offers a small molecular building block for the development of enzyme based molecular self-assembly. Furthermore, *cis*/*trans*-isomerization offers a novel pathway for achieving well-dispersed nanofibers, which could be used in drug separation, drug release and other fields.

## Supporting Information

File 1 Experimental part.
