# Supplementary material for: Exceptionally small supramolecular hydrogelators based on aromatic–aromatic interactions
Source: Beilstein J Org Chem. 2011 Feb 7;7:167–72. doi: 10.3762/bjoc.7.23 (PMC3063069; doi:10.3762/bjoc.7.23)
Supplement: File 1 — Experimental part. [file Beilstein_J_Org_Chem-07-167-s001.pdf]

Supporting Information

for

Exceptionally small supramolecular hydrogelators based on aromatic–aromatic interactions

Junfeng Shi[1], Yuan Gao[1], Zhimou Yang[2] and Bing Xu*[1]

Address: [1]Department of Chemistry, Brandeis University, 415 South Street, Waltham, Massachusetts 02453 and [2]The Key Laboratory of Bioactive Materials, Ministry of Education, College of Life Science, NanKai University, Tianjin 300071, People's Republic of China

E-mail: Bing Xu* - bxu@brandeis.edu

*Corresponding author

Experimental part

Materials

Chemical reagents and solvents were used as received from commercial sources. ^1H NMR spectra were obtained on Varian Unity Inova 400 spectrometer, Fluorescence spectra on RF5301PC fluorescence spectroscopy, TEM on Morgagni 268 transmission electron microscope, rheological data on ARES G2 rheometer.

Synthesis

Synthesis of **3**: synthesis of **3** was described in the literature [1]. Briefly, 2-(naphthalen-6-yl)acetic acid (372 mg 2 mmol) and *N*-hydroxysuccinimide (NHS) (230 mg, 2 mmol) were dissolved in 40 mL of chloroform, and N-dicyclohexylcarbodiimide (DCC) (453 mg, 2.2 mmol) and 4-dimethylaminopyridine (DMAP) (13 mg, 0.1 mmol)

were added to the above solution with stirring. After the reaction mixture was stirred at room temperature for 1.5 h, the resulting solid was filtered off and the filtrate was evaporated in an open dish to dryness and the crude product was used in the next reaction without purification.

L-Phenylalanine (332 mg, 2 mmol) and $NaHCO_3$ (336 mg, 4 mmol) were dissolved in 10 mL of water with stirring; the crude product was dissolved in acetone, and dropped into above water solution. If the mixture become not clear, added in extra acetone to make it clear, and the resulting reaction mixture was stirred at room temperature for 24 h. The reaction mixture was air-dried, then 30 mL of water was added and the precipitate was removed by filtration. The filtrate was acidified to pH = 2～3 and the resulting product was obtained by filtration, washed with water, and then dried in vacuum. ^{1}H NMR (DMSO, 400 MHz): δ = 2.8-3.2 (2H Ar-H), 3.58 (1H, CHCOO), 4.4 (2H, $COCH_2$), 7.0-8.0 (12H, ArH). ^{13}C NMR (100 MHz): δ = 36.79, 42.18, 53.68, 125.52, 126.06, 126.39, 127.27, 127.39, 127.51, 127.63, 128.13, 129.22, 131.76, 132.95, 133.95, 137.69, 169.97, 173.12. mp: 129-130 °C

Synthesis of **6**: Cinna-Cl (cinnamoyl chloride) (166 mg, 1.0 mmol) was dissolved in 5 mL dioxane and L-phenylalanine (Phe,165 mg, 1.0 mmol) was dissolved in 15 mL 10% (w/w) Na_2CO_3 solution. The Cinna-Cl solution was added dropwise into the Phe solution. The reaction was conducted in an icy water bath. After 1 h, the icy water bath was removed and the reaction continued for 2 h at ambient temperature. Then the solution was diluted with 60 mL water. The mixture was washed with ether two times (20 mL each) and the aqueous phase was adjusted to pH = 1.0 with 37% hydrochloric acid. The acidic aqueous layer was extracted with ethyl acetate three times (20 mL each). The organic layer was combined and evaporated under reduced pressure to remove solvent. The resultant product was dried under vacuum overnight. ^{1}H NMR (DMSO, 400 MHz): δ = 2.8-3.2 (2H, Ar-H), 4.57 (1H, CHCOO), 6.6-6.8 (1H, RC=CH), 7.34-7.44 (1H, HC=CR), 7.0-7.7 (10H, ArH), 8.3-8.6 (1H, CONH). ^{13}C NMR (100 MHz): δ = 36.96, 53.84, 121.76, 126.61, 127.71, 128.38, 129.11, 129.23, 129.71, 134.88, 137.75, 139.39, 165.02, 173.20. mp: 146-147 °C

Data for **4**: ^{1}H NMR (DMSO, 400 MHz): δ = 2.9-3.2 (1H, Ar-H), 4.51 (1H, CHCOOH), 4.59 (2H, OCH_2), 7.0-8.0 (12H, ArH). ^{13}C NMR (100 MHz): δ = 36.60, 53.47, 66.86, 107.49, 118.74, 124.06, 126.57, 126.62, 126.98, 127.70, 128.34, 128.92, 129.31, 129.52, 134.21, 137.82, 155.67, 167.75, 173.02. mp: 172-173 °C
Compound **1** was purchased from Sigma, compound **2** and **4** was synthesized according to the procedure of **3**, and compound **5** was obtained by following the procedure of **6**.

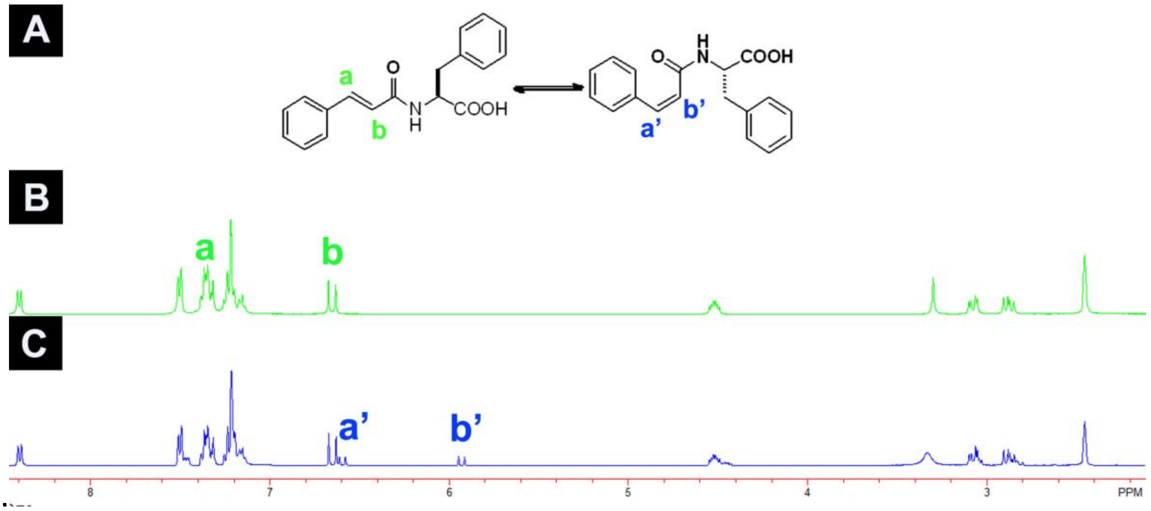

Figure S1: (A) the structure of compound **6** in *trans*- and *cis*-isomers**,** (B) the NMR spectra of compound **6** before and (C) after UV irradiation for 2 hours.

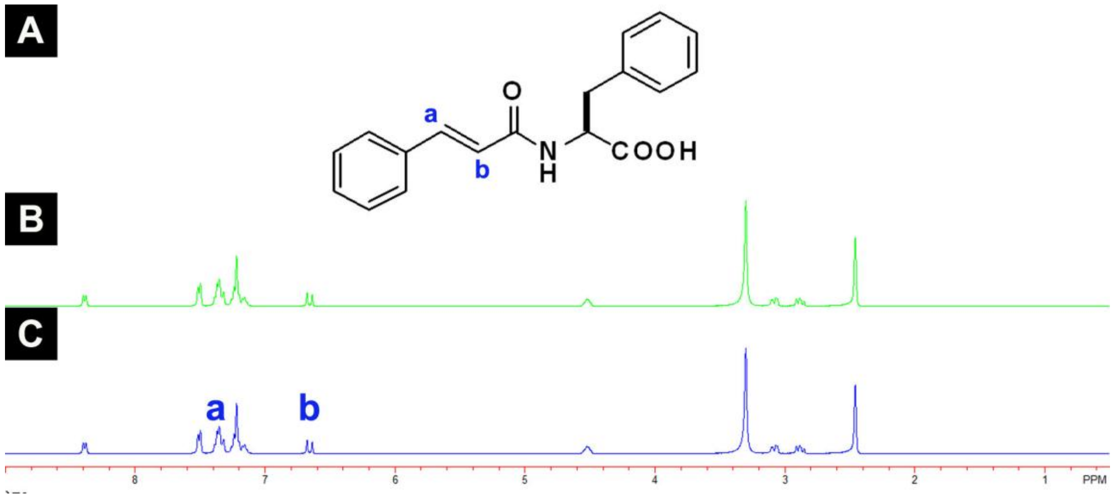

Figure S2: (A) The structure of compound **6** in trans-isomer, (B) the NMR spectra of compound **6** after and (C) before heating at 85 °C for 2 hours.

Reference

(1) Yang, Z. M.; Gu, H. W.; Du, J.; Gao, J. H.; Zhang, B.; Zhang, X. X.; Xu, B. *Tetrahedron* **2007**, *63*, 7349.
